# Complete Genome Sequence of *Sulfitobacter* sp. Strain D7, a Virulent Bacterium Isolated from an *Emiliania huxleyi* Algal Bloom in the North Atlantic

**DOI:** 10.1128/MRA.01379-18

**Published:** 2018-11-15

**Authors:** Chuan Ku, Noa Barak-Gavish, Mark Maienschein-Cline, Stefan J. Green, Assaf Vardi

**Affiliations:** a Department of Plant and Environmental Sciences, Weizmann Institute of Science, Rehovot, Israel; b Research Informatics Core, Research Resources Center, University of Illinois at Chicago, Chicago, Illinois, USA; c Sequencing Core, Research Resources Center, University of Illinois at Chicago, Chicago, Illinois, USA; Louisiana State University

## Abstract

A *Rhodobacterales* bacterium, *Sulfitobacter* sp. strain D7, was isolated from an Emiliania huxleyi bloom in the North Atlantic and has been shown to act as a pathogen and induce cell death of E. huxleyi during lab coculturing. We report here its complete genome sequence comprising one chromosome and five low-copy-number plasmids.

## Abstract (precis)

A *Rhodobacterales* bacterium, *Sulfitobacter* sp. strain D7, was isolated from an Emiliania huxleyi bloom in the North Atlantic and has been shown to act as a pathogen and induce cell death of E. huxleyi during lab coculturing.

## ANNOUNCEMENT

The coccolithophore Emiliania huxleyi (Haptophyta) is a cosmopolitan marine microalga that plays an important role in global carbon and sulfur cycling by forming massive blooms and producing copious amounts of dimethylsulfoniopropionate (DMSP) and the atmospherically active compound dimethyl sulfide (1). Here, we report the complete genome sequence of *Sulfitobacter* sp. strain D7, a *Rhodobacterales* bacterium that, when cocultured with E. huxleyi, causes death of the alga in a DMSP-dependent manner (2). It was isolated from the microbiome of copepods collected during a natural E. huxleyi bloom in the North Atlantic, and its cooccurrence with the alga in the water column was confirmed by quantitative PCR (qPCR) (2).

Genomic DNA was extracted from a culture grown overnight in 1/2 yeast extract-tryptone-Sigma sea salts (YTSS) medium at 28°C under agitation (150 rpm) using the DNeasy blood and tissue kit (Qiagen, Hilden, Germany). A library with an insert size of ∼500 bp was prepared using the Nextera XT kit (Illumina, San Diego, CA) and sequenced using the Illumina NextSeq 500 platform to generate 150-bp paired-end reads. A library of fragments longer than 10 kb was prepared following the PacBio (Menlo Park, CA) protocol, loaded with MagBeads, and sequenced on the RS II platform. Hybrid assembly of both PacBio (*N*_50_, 10,282 bp) and Illumina reads was performed using the software package SPAdes v. 3.11 (3) with “31,51,71,91” for k-mers. After filtering for contig size (>500 bp) and Illumina read coverage (at least one-third of the coverage level at half the total assembly length) based on Bowtie2 v. 2.3.0 (4) mapping, six contigs longer than 80 kb were retained from the initial draft. Sequence errors, misassemblies, and gaps were corrected by mapping Illumina reads to the contigs with the Burrows-Wheeler Aligner (BWA) v. 0.7.12 (5) and visual inspection in Integrative Genomics Viewer (IGV) v. 2.3.90 (6), as in a previous study (7). The assembly was also checked by mapping the PacBio reads using Basic Local Alignment with Successive Refinement (BLASR) v. 5.3 (8) with the default settings for RS II reads. Completion and circularization of each contig were confirmed by Illumina read pairs and PacBio reads that joined the two ends. The NCBI Prokaryotic Genome Annotation Pipeline v. 4.1 (9) was used for gene predictions and annotations.

The complete genome sequence consists of six circular replicons (Table 1), including one 3,371,091-bp chromosome with an average GC content of 61.4% and five plasmids of between 81 and 193 kb that show low or single copy numbers characteristic of *Rhodobacterales* and other alphaproteobacteria (10, 11). In total, there are 4 complete rRNA operons, 47 tRNAs, 3,744 protein-coding genes, and 52 pseudogenes, with all RNA genes located on the chromosome. Consistent with observation of the production of methanethiol, a metabolic by-product of DMSP, during coculturing with E. huxleyi, the chromosome encoded *dmdA* for demethylation of DMSP (2). In addition, we identified type I and type II secretion system genes on the chromosome and type IV genes on the plasmids p2SUD7 and p3SUD7, which may be involved in the bacterium’s interactions with the alga.

**TABLE 1:** Features and accession numbers of the *Sulfitobacter* sp. D7 genome sequence

Replicon	GenBank accession no.	Size (bp)	GC content (%)	Illumina read coverage (fold)
Chromosome	CP020694	3,371,091	61.4	918
p1SUD7	CP020695	193,276	62.3	585
p2SUD7	CP020696	126,656	58.5	382
p3SUD7	CP020697	104,922	59.9	351
p4SUD7	CP020698	85,454	57.5	778
p5SUD7	CP020699	81,709	58.9	405

### Data availability.

The complete genome sequence of *Sulfitobacter* sp. strain D7 has been deposited in GenBank under the accession numbers CP020694 to CP020699. Raw Illumina and PacBio RS II sequence reads have been deposited in the NCBI Sequence Read Archive under the accession numbers SRR7948486 and SRR7948487, respectively.
